# *In Vitro* Susceptibility and Resistance of Mycoplasma genitalium to Nitroimidazoles

**DOI:** 10.1128/aac.00006-23

**Published:** 2023-03-09

**Authors:** Gwendolyn E. Wood, Caroline M. Kim, Laarni Kendra T. Aguila, Robert H. Cichewicz

**Affiliations:** a Department of Medicine, Division of Allergy and Infectious Diseases, University of Washington, Seattle, Washington, USA; b Department of Chemistry and Biochemistry, University of Oklahoma, Norman, Oklahoma, USA; c Natural Products Discovery Group, University of Oklahoma, Norman, Oklahoma, USA; d Institute for Natural Products Applications and Research Technologies, University of Oklahoma, Norman, Oklahoma, USA

**Keywords:** *Mycoplasma genitalium*, antibiotic resistance, metronidazole, nitroimidazole, secnidazole, tinidazole

## Abstract

Mycoplasma genitalium is a sexually transmitted reproductive tract pathogen of men and women. M. genitalium infections are increasingly difficult to treat due to poor efficacy of doxycycline and acquired resistance to azithromycin and moxifloxacin. A recent clinical trial suggested that metronidazole may improve cure rates for women with pelvic inflammatory disease and reduced the detection of M. genitalium when included with standard doxycycline plus ceftriaxone treatment. As data regarding susceptibility of mycoplasmas to nitroimidazoles are lacking in the scientific literature, we determined the *in vitro* susceptibility of 10 M. genitalium strains to metronidazole, secnidazole, and tinidazole. MICs ranged from 1.6 to 12.5 μg/mL for metronidazole, 3.1 to 12.5 μg/mL for secnidazole, and 0.8 to 6.3 μg/mL for tinidazole. None of these agents was synergistic with doxycycline in checkerboard broth microdilution assays. Tinidazole was superior to metronidazole and secnidazole in terms of MIC and time-kill kinetics and was bactericidal (>99.9% killing) at concentrations below reported serum concentrations. Mutations associated with nitroimidazole resistance were identified by whole-genome sequencing of spontaneous resistant mutants, suggesting a mechanism for reductive activation of the nitroimidazole prodrug by a predicted NAD(P)H-dependent flavin mononucleotide (FMN) oxidoreductase. The presence of oxygen did not affect MICs of wild-type M. genitalium, but a nitroimidazole-resistant mutant was defective for growth under anaerobic conditions, suggesting that resistant mutants may have a fitness disadvantage in anaerobic genital sites. Clinical studies are needed to determine if nitroimidazoles, especially tinidazole, are effective for eradicating M. genitalium infections in men and women.

## INTRODUCTION

Mycoplasma genitalium is a sexually transmitted pathogen of the genital tract of men and women. In men, M. genitalium is a common cause of nongonococcal urethritis (1); in women it is associated with cervicitis, pelvic inflammatory disease (PID), infertility, and preterm birth (2). Treatment of M. genitalium infection is becoming increasingly difficult due to the rapid increase in resistance to azithromycin and moxifloxacin, which serve as front-line therapies. Globally, more than 50% of M. genitalium strains carry macrolide resistance mutations, and fluoroquinolone resistance exceeds 10% (3). High-risk populations have even higher rates of resistance, and dual class resistance is increasing in the United States and worldwide (3, 4). Resistance-guided therapy, now recommended by the U.S. Centers for Disease Control and Prevention, involves the syndromic treatment of suspected M. genitalium infections with doxycycline to reduce organism load while molecular testing for the organism and macrolide resistance mutations is performed (5, 6). Subsequently, macrolide-susceptible infections are treated with azithromycin while macrolide-resistant infections receive moxifloxacin. However, macrolide detection assays are currently not commercially available in the United States, limiting resistance-guided therapy to research centers. Additional treatment options for M. genitalium infections are needed, especially as moxifloxacin is associated with serious side effects, is contraindicated in pregnancy, and is not approved for use in adolescents.

Treatment guidelines for PID recommend antimicrobials that target Neisseria gonorrhoeae, Chlamydia trachomatis, and anaerobic organisms (6). A recent randomized controlled trial by Wiesenfeld et al. (7) found that adding metronidazole to standard doxycycline-plus-ceftriaxone treatment regimens improved treatment outcomes. Unexpectedly, the addition of metronidazole also reduced the incidence of M. genitalium (14 versus 4%) in women with PID, leading to the hypothesis that anaerobic bacteria enhance colonization by M. genitalium (7, 8).

Here, we assessed the *in vitro* susceptibility of M. genitalium to nitroimidazoles including metronidazole, secnidazole, and tinidazole. We found that these agents have direct *in vitro* activity against 10 strains of M. genitalium with MICs similar to or below serum concentrations with standard dosing regimens. In addition, we identified mutations present in nitroimidazole-resistant mutants, plausibly suggesting a mechanism of reductive activation of the nitroimidazole prodrug. Our findings suggest that nitroimidazoles, especially tinidazole, may represent a promising addition to the short list of FDA-approved antimicrobials with activity against this organism.

## RESULTS

### *In vitro* susceptibility assays.

**FIG 1:**
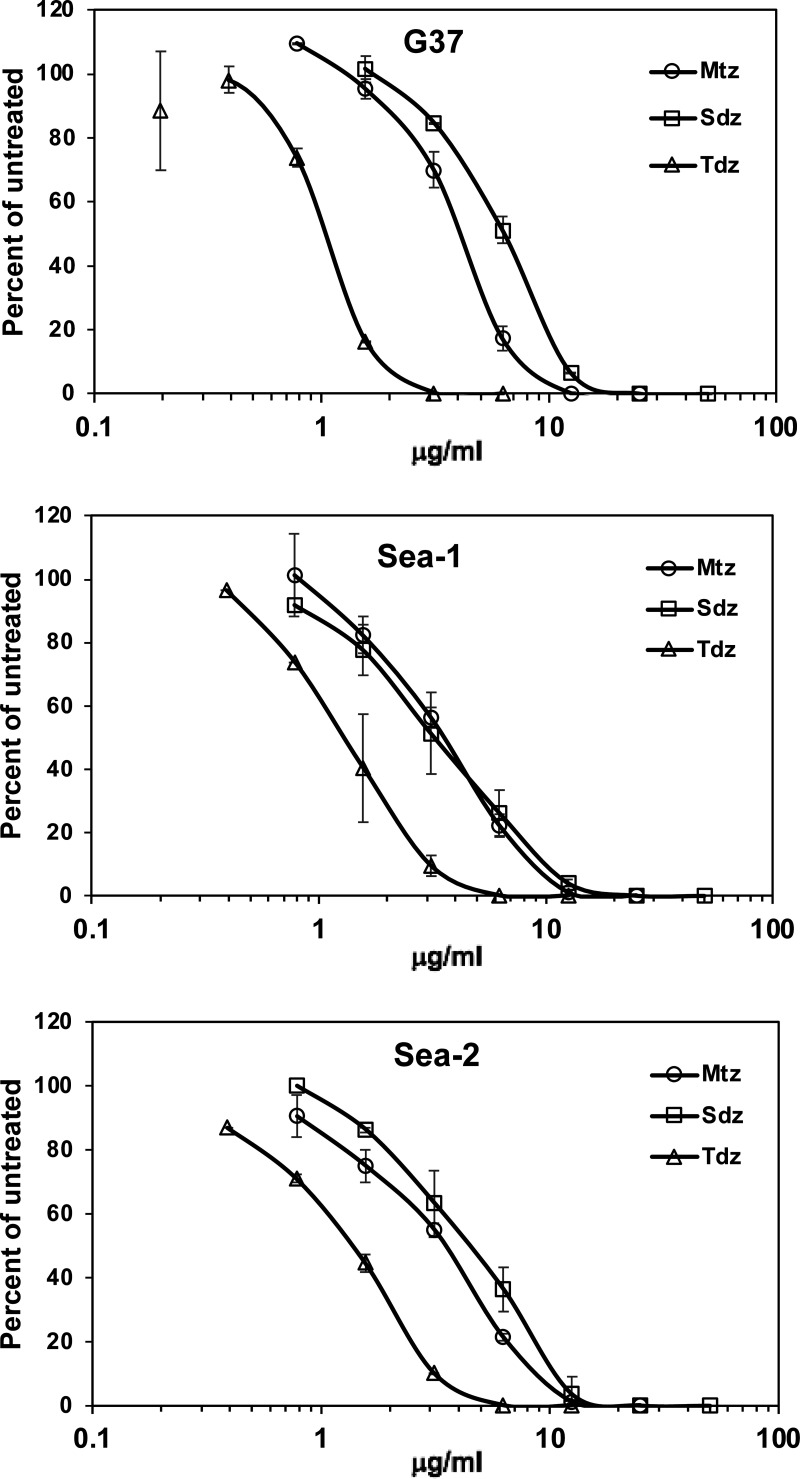
Growth inhibition dose response of M. genitalium strains G37, Sea-1, and Sea-2 to nitroimidazoles. Mtz, metronidazole; Sdz, secnidazole; Tdz, tinidazole. The *y* axis shows the mean number of genomes detected in triplicate qPCR measurements compared to untreated M. genitalium. Errors bars show standard deviation for duplicate drug-treated wells. The experiment was repeated three times with similar results.

*In vitro* susceptibility testing was performed using three commercially available nitroimidazoles (metronidazole, secnidazole, and tinidazole) against five M. genitalium strains adapted to axenic growth in broth microdilution assays. The MICs were 6.3 to 12.5 μg/mL for metronidazole, 12.5 μg/mL for secnidazole, and 3.1 μg/mL for tinidazole for these axenic strains (Table 1). As broth microdilution assays rely on the somewhat subjective interpretation of color change, and no CLSI standards exist for M. genitalium susceptibility testing, we used quantitative PCR (qPCR) to quantify inhibition for three axenic strains. As shown in Fig. 1, M. genitalium strains G37, Sea-1, and Sea-2 exhibited a concentration-dependent response to each nitroimidazole and50% inhibitory concentration (IC_50_) values confirmed that tinidazole was more potent than metronidazole or secnidazole for all three strains (Table 2). The susceptibility of five additional M. genitalium strains isolated from men with urethritis in Seattle, WA (9), and dependent on Vero cell coculture, was determined using a qPCR method. The Vero cell coculture assay confirmed the MIC values obtained in broth microdilution assays for strain G37, and MICs for comparator antibiotics (doxycycline, moxifloxacin, and azithromycin) were similar to previously published results (9), thereby validating assay performance. Considering all 10 strains, MICs were lowest for tinidazole, ranging from 0.8 to 6.3 μg/mL (MIC_50_ = 3.1 μg/mL, MIC_90_ = 3.1 μg/mL), followed by metronidazole (MIC = 6.3 to 12.5 μg/mL, MIC_50_ = 6.3 μg/mL, MIC_90_ = 12.5 μg/mL), and secnidazole (MIC = 3.1 to 12.5 μg/mL, MIC_50_ = 12.5 μg/mL, MIC_90_ = 12.5 μg/mL).

**TABLE 1:** *In vitro* susceptibility of M. genitalium strains

Method and strain	MIC (μg/mL) of drug					
	Metronidazole	Secnidazole	Tinidazole	Doxycycline	Moxifloxacin	Azithromycin
Broth microdilution^*a*^						
G37	6.3	12.5	3.1	0.25	0.125	0.008
Sea-1	6.3	12.5	3.1	ND^*b*^	ND	ND
Sea-2	12.5	12.5	3.1	ND	ND	ND
M2282	6.3	12.5	3.1	0.5	0.25	<0.0025
M2300	6.3	12.5	3.1	0.125	0.125	<0.0025
Vero cell coculture^*c*^						
G37	6.3	12.5	3.1	0.5	0.125	ND
MEGA 601	6.3	12.5	3.1	0.25^*d*^	0.25	<0.0025^*d*^
MEGA 1082	1.6	3.1	0.8	0.25	>1	>8
MEGA 1166	12.5	12.5	0.8	0.125^*d*^	<0.031	<0.0025^*d*^
MEGA 1256	6.3	6.3	3.1	0.125^*d*^	0.125	>8^*d*^
MEGA 1272	6.3	12.5	6.3	0.5^*d*^	0.063	>8^*d*^

a MIC defined as lowest concentration with no color change.

b ND, not done.

c MIC defined as the concentration inhibiting growth by 99% compared to untreated M. genitalium as determined by qPCR.

d These MICs were also published previously (9).

**TABLE 2:** IC_50_ values for nitroimidazoles against M. genitalium^*a*^

Strain	IC_50_ (μg/mL) of drug:		
	Metronidazole	Secnidazole	Tinidazole
G37	3.81 ± 0.33	6.06 ± 0.05	1.12 ± 0.25
Sea-1	3.30 ± 0.85	3.45 ± 1.50	1.20 ± 0.61
Sea-2	4.41 ± 0.50	5.71 ± 1.09	1.70 ± 0.08

a Values are the means for duplicate wells ± standard deviation.

### Synergism with doxycycline.

Synergy of metronidazole, secnidazole, and tinidazole in combination with doxycycline was measured by checkerboard broth microdilution assay using M. genitalium strain G37. When combined with doxycycline, the MICs of each nitroimidazole were reduced ~2-fold and doxycycline MICs were reduced ~4-fold compared to values in Table 1. This resulted in a fractional inhibitory concentration index (FICI) of 0.75 for each nitroimidazole in combination with doxycycline, indicating either no interaction according to the criteria of Odds (10) or an additive effect using the criteria of Doern (11).

### Time-kill assays.

**FIG 2:**
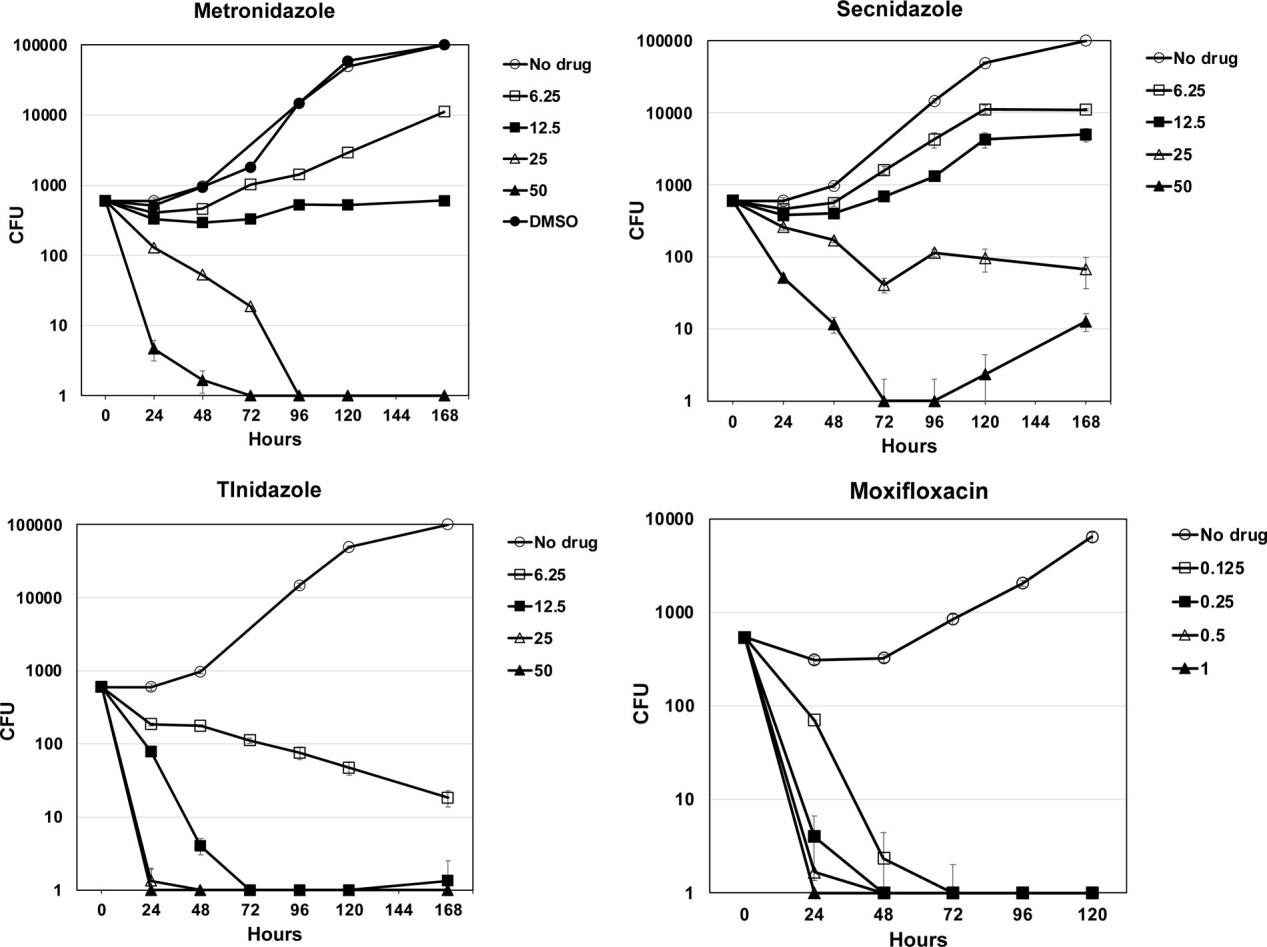
Time-kill experiments. Killing curves for M. genitalium strain G37 treated with various concentrations (micrograms per milliliter) of metronidazole, secnidazole, tinidazole, or moxifloxacin over time. Curves show the average CFU per 10-μL aliquot spotted in triplicate on SP-4 agar plates. Error bars show standard deviation. DMSO, solvent control, is shown in the upper left panel. Results of a typical experiment repeated twice are shown.

To explore the kinetics of M. genitalium killing by nitroimidazoles, time-kill assays were performed using doubling dilutions of nitroimidazoles spanning 50 to 6.3 μg/mL in SP-4 broth (Fig. 2). Metronidazole was bactericidal (>99.9% killing) at 25 μg/mL after 96 h and at 50 μg/mL after 72 h. Killing by secnidazole was less efficient: >99.9% killing occurred at 50 μg/mL after 72 h and regrowth of ~1 log_10_ CFU was observed after 5 to 7 days of exposure to 50 μg/mL secnidazole (Fig. 2). Tinidazole was most active, with bactericidal effects after 72 h at 12.5 μg/mL, 48 h at 25 μg/mL, and 24 h at 50 μg/mL. Moxifloxacin, included for comparison in these experiments (Fig. 2), was bactericidal after 24 to 72 h at 0.125 to 1 μg/mL.

### Nitroimidazole resistance.

The rate of spontaneous resistance to nitroimidazoles was estimated by plating M. genitalium strain G37S (12) on SP-4 agar plates containing antibiotic (100 μg/mL). The spontaneous resistance rates were similar for the three nitroimidazoles: 2.18 × 10^−7^, 3.93 × 10^−7^, and 1.72 × 10^−7^ for metronidazole, secnidazole, and tinidazole, respectively.

Four spontaneous nitroimidazole-resistant mutants were isolated and characterized. Two mutants, MtzR(A) and MtzR(B), were selected by serial passage in increasing concentrations of metronidazole (10 to 50 μg/mL) and then single-colony cloning. Mutants MtzR(E) and TdzR(A) were isolated after plating directly on SP-4 agar plates containing 50 μg/mL metronidazole or tinidazole, respectively. Broth microdilution assays confirmed that each of these mutant strains was cross-resistant to ≥100 μg/mL of metronidazole, secnidazole, and tinidazole.

**FIG 3:**
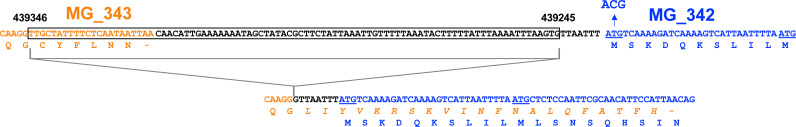
Nitroimidazole resistance-associated mutations in the *MG_342-MG_343* intergenic region. For clarity, the M. genitalium genome sequence is shown in reverse orientation (note genome coordinates). The large gray triangle indicates the deletion of 92 bp (boxed) in MtzR(A) that changes the last 6 amino acids of *MG_343* to 21 missense amino acids (indicated by italics) and overlaps the MG342 start codon. Possible ATG start codons are underlined. The T-to-C base change in the *MG_342* start codon of mutants MtzR(E) and TdzR(A) is indicated above the sequence (small arrow). The W93L mutation in *MG_342* of MtzR(B) is not shown.

Whole-genome sequencing of these four mutants using Oxford Nanopore sequencing technology revealed that each had a mutation in or near the *MG_342* gene, annotated as an NAD(P)H-dependent flavin mononucleotide (FMN) oxidoreductase. Mutant MtzR(A) contained two mutations: (i) a 92-bp deletion encompassing bp 439245 to 439346 and (ii) a single base change at bp 481520 (see below). The 92-bp deletion predicts the replacement of the last 6 amino acids of MG343 (annotated as a conserved hypothetical protein) with 21 missense amino acids and deletes 70 bp upstream of *MG_342* (Fig. 3). Translation of MG342 may be affected in this mutant as the MG343 C terminus now overlaps the native start codon of *MG_342* (Fig. 3); a second ATG located 30 bp downstream may function as an alternative start codon. In addition, deletion of 92 bp in this mutant could affect transcription of *MG_342* by removing a potential promoter sequence in the *MG_343-MG_342* intergenic region.

Mutants MtzR(E) and TdzR(A) both have a single A-to-G mutation at bp 439235 which changes the *MG_342* start codon from ATG to ACG on the coding strand. Again, the ATG located 30 bp downstream may serve as the translational start codon for MG342 in these mutants.

**FIG 4:**
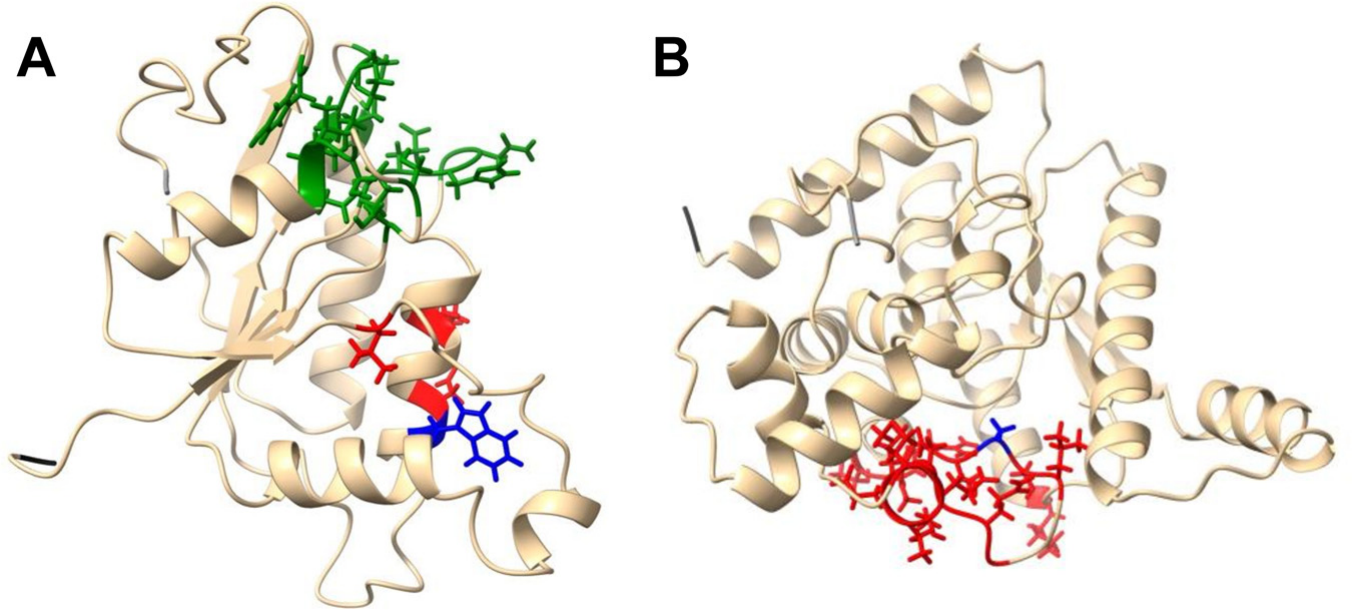
(A) Model of MG342. FMN binding (green) and active site (red) residues and Trp93 (blue) are shown. C score = 0.39; TM score = 0.77 ± 0.10; root mean square deviation (RMSD) = 4.2 ± 2.8 Å. (B) Model of MG383. Active site residues are shown in red, and Ala186 (involved in NAD binding) is shown in blue. C score = 0.43; TM score = 0.77 ± 0.10; RMSD = 4.2 ± 2.8 Å. Both proteins were modeled with I-TASSER. N and C termini are indicated in dark and light gray, respectively. Images were produced with UCSF ChimeraX (version 1.3).

Sequencing of metronidazole-resistant mutant MtzR(B) identified a C-to-A point mutation (G to T on the coding strand) at bp 438959 within *MG_342*, predicting a Trp93Leu amino acid change. Alignments of homologs identified by BLAST show that MG342 is 73% identical to Mycoplasma pneumoniae MPN517 (new locus tag FA921_RS02955), 36 to 37% identical to homologs in other *Mycoplasma* species (*M. alvi*, *M. pirum*, *M. testudinis*, and *M. imitans*), and 35 to 36% identical to homologs in other bacteria (Psychromonas ingrahamii, *Vibrio* species, Pseudoalteromonas shioyasakiensis, and *Moritella* sp.). Trp93 is 100% conserved in all homologs of MG342. BLAST failed to identify homologs of MG342 in Mycoplasma hominis or *Ureaplasma* sp. Modeling of the MG342 protein sequence with I-TASSER (13–15) suggested a structure similar to that of other oxidoreductases, identified putative FMN binding residues (confidence score of 0.77), and located Trp93 near predicted active site residues (Fig. 4A). In addition, vector alignment search tool (VAST) (16) identified proteins with similar structures (*P* < 10^−6^), including azoreductases that activate nitrofuran drugs through nitroreductase activity (17). Given its homology with nitroreductases we hypothesize that MG342 participates in the reductive activation of the nitroimidazole prodrug which is necessary for its antimicrobial activity. As *MG_342* is likely an essential gene (18, 19), we speculate that the mutations in our resistant strains reduce, but do not abolish, MG342 expression and/or enzymatic activity.

In addition to mutations in *MG_342*, resistant mutant MtzR(A) also contained a single base change (G to A) at bp 481520, predicting an Ala186Thr mutation in MG383. *MG_383* is annotated as a putative NH_3_-dependent NAD^+^ synthetase (NadE) and is reported to be an essential gene (18, 19). An NCBI conserved domain search and modeling with I-TASSER predicted that Ala186 lies within the NAD binding active site (Fig. 4B). Whether this conservative Ala186Thr mutation affects MG383 function is not known. Other than those noted above, no other sequence changes were found in the genomes of the four nitroimidazole-resistant mutants. Together, these results suggest that resistance to nitroimidazoles is conferred by mutations in the *MG_342* gene, although mutations in *MG_383* may also play a role.

### Effect of oxygen tension nitroimidazole susceptibility.

The effect of oxygen on nitroimidazole susceptibility was determined by incubating broth microdilution assay mixtures in microaerophilic and anaerobic atmospheres. As shown in Table 3, nitroimidazole MICs for wild-type M. genitalium strain G37 were similar under aerobic, microaerophilic, and anaerobic conditions, suggesting that oxygen has no impact on the reductive activation of nitroimidazoles in this organism. The MICs for the nitroimidazole-resistant mutant MtzR(A) were >100 μg/mL under both microaerophilic and aerobic conditions. Surprisingly, this strain failed to grow under anaerobic conditions, suggesting that nitroimidazole resistance may confer a fitness disadvantage under low-oxygen conditions.

**TABLE 3:** Effect of atmosphere on nitroimidazole MICs of susceptible and resistant M. genitalium strains

Drug	MIC (μg/mL) for strain under condition:					
	Aerobic^*a*^		Microaerophilic^*b*^		Anaerobic^*c*^	
	G37C	MtzR(A)	G37C	MtzR(A)	G37C	MtzR(A)
Metronidazole	6.3	>100	12.5	>100	6.3	No growth^*d*^
Secnidazole	12.5	>100	25	>100	6.3	No growth
Tinidazole	3.1	100	6.3	100	3.1	No growth
Moxifloxacin	0.125	0.125	0.125	0.125	0.125	No growth
Doxycycline	0.2	0.2	0.4	0.2	0.4	No growth

a Aerobic = 5% CO_2_, 95% air (~20% O_2_), 7 days of incubation.

b Microaerophilic = candle jar; 3 to 5% CO_2_, 8 to 10% O_2_, 7 days of incubation.

c Anaerobic = AnaeroGen system; 8 to 14% CO_2_, <1% O_2_, 15 days of incubation.

d Strain MtzR(A) did not grow during 15 days of incubation.

## DISCUSSION

In this study, we investigated the *in vitro* activity of metronidazole, secnidazole, and tinidazole against 10 strains of M. genitalium (including broth-adapted and Vero coculture-dependent clinical isolates). MICs ranged from 1.6 to 12.5 μg/mL for metronidazole (MIC_50_ = 6.3 μg/mL and MIC_90_ = 12.5 μg/mL), 3.1 to 12.5 μg/mL for secnidazole (MIC_50_ and MIC_90_ = 12.5 μg/mL), and 0.8 to 6.3 μg/mL for tinidazole (MIC_50_ and MIC_90_ = 3.1 μg/mL). In addition, time-kill experiments demonstrated that of these three agents, tinidazole is most active: tinidazole was bactericidal (>99.9% killing) after 72 h at 12.5 μg/mL or 48 h at 25 μg/mL. Metronidazole was less effective, requiring 96 h at 25 μg/mL or 72 h at 50 μg/mL. Secnidazole killed ≥99.9% after 72 h at 50 μg/mL.

Although mycoplasmas are commonly described as resistant to nitroimidazoles, very few published data are available to substantiate these claims. Two studies found that metronidazole had no impact on Ureaplasma urealyticum infection in women with bacterial vaginosis: *U. urealyticum* persisted in 63/64 women with nonspecific vaginitis (20), and detection was unchanged in 34 women before and after metronidazole treatment (20/34 versus 22/34) (21). Limited *in vitro* data indicate that Mycoplasma hominis is resistant to metronidazole (MICs of ≥64 μg/mL, determined under anaerobic conditions), but only three isolates were tested (22). Despite these high MICs, metronidazole treatment reduced *M. hominis* infection in several studies of women with bacterial vaginosis (20–22), suggesting either that strains vary in their susceptibility or that clearance is due to effects on other organisms with which *M. hominis* coinfects. For example, among women with nonspecific vaginitis 18/47 (38%) metronidazole-treated women had persistent *M. hominis* compared to 16/23 (70%) women treated with ampicillin (22). Persistence of *M. hominis* was specifically associated with the presence of *Bacteroides* species after metronidazole treatment, suggesting a synergistic association of these two organisms with these genital tract syndromes (22). Defining the activity of metronidazole against *M. hominis* is further complicated by conflicting reports regarding the ability of this organism to affect the metronidazole susceptibility of Trichomonas vaginalis, a protozoan parasite that can host intracellular infection with *M. hominis* (23).

Recent clinical data suggest that nitroimidazoles may have *in vivo* activity against M. genitalium. Schwebke et al. (24) found that the addition of tinidazole (single 2-g dose to target Trichomonas vaginalis) to doxycycline or azithromycin for treatment of nongonococcal urethritis decreased the prevalence of M. genitalium from 76% to 61% and from 41% to 26%, respectively. Although neither result was statistically significant (*P* = 0.24 and 0.19, respectively), the trend toward efficacy against M. genitalium is consistent with the pharmacokinetics of tinidazole: serum levels reach 40 to 58 μg/mL after a 2-gram dose, falling to 10 to 14.5 μg/mL in 24 h, which is insufficient for >99.9% killing but may be effective in some patients. A more recent study by Wiesenfeld et al. (7) reported that cervical M. genitalium was significantly (*P* < 0.05) reduced in women with PID when metronidazole was added to the standard therapy of doxycycline plus ceftriaxone (from 14.7 to 4.4%) compared to doxycycline plus ceftriaxone with placebo (20.5 to 14.1%). Doxycycline is 30 to 45% effective (25), and the additional clearance afforded by metronidazole is consistent with our *in vitro* data as the metronidazole dose used (500 mg twice daily for 14 days) produces average serum concentrations (12 to 18 μg/mL [26]) that are sufficient to inhibit growth but not kill >99.9% of M. genitalium isolates.

Nitroimidazoles were first recognized as potent antimicrobials for anaerobic bacteria but are now known to have activity against microaerophilic bacteria. Nitroimidazoles enter the cell as an inactive prodrug and are then reductively activated by a variety of mechanisms (27). One mode of activation involves nitroreductases that contain flavin adenine dinucleotide (FAD)/FMN prosthetic groups and use NADH/NADPH as reducing agents (28). M. genitalium mutants resistant to high levels of nitroimidazoles had mutations in or near *MG_342*, encoding a predicted NAD(P)H-dependent FMN oxidoreductase, leading to the hypothesis that this protein functions in the reductive activation of nitroimidazoles. Two mutants had changes in the start codon (ATG to ACG), predicting reduced expression of MG342, as ACG does not serve as a start codon in any *Mycoplasma* genomes (29) and is expressed at <0.1% of ATG in Escherichia coli (30). Another mutant had a deletion immediately upstream of *MG_342*, which may affect the activity of the upstream gene product (unknown function) and/or the expression of MG342. As *MG_342* is reportedly an essential gene, we hypothesize that an ATG located 30 bp downstream functions as the start codon to allow sufficient expression for growth of these mutants. A fourth mutant had a mutation in Trp93, which is located near the predicted active site of MG342 (and conserved in all homologs of MG342 identified by BLAST) and may reduce MG342 activity. Interestingly, *M. hominis* and *Ureaplasma* species lack orthologs of *MG_342* (31). Future work will test our hypothesis that MG342 activates nitroimidazoles in M. genitalium.

Nitroimidazole susceptibility in some organisms is affected by the presence of oxygen (27). For example, metronidazole MICs of susceptible and resistant strains of Helicobacter pylori are lower under anaerobic than microaerophilic growth conditions (32), and Mycobacterium tuberculosis is more susceptible to metronidazole under anaerobic conditions (33). M. genitalium is a facultative anaerobe that was first isolated under anaerobic conditions (34) but is routinely cultured under aerobic conditions, allowing the comparison of nitroimidazole MICs under different oxygen atmospheres. We found that oxygen had no effect on nitroimidazole MICs for wild-type M. genitalium, suggesting that activation of nitroimidazoles occurs via an oxygen-insensitive mechanism. Interestingly, a nitroimidazole-resistant mutant failed to grow under anaerobic conditions, implying that nitroimidazole resistance confers a fitness cost. We are currently investigating whether this anaerobic growth defect is common among other nitroimidazole-resistant mutants.

In summary, nitroimidazoles represent a promising additional therapy for M. genitalium treatment, especially as they are widely available and extensive clinical studies to assess dosing and safety have already been done. M. genitalium is one of three organisms added to the CDC Watch List of antibiotic resistance threats in the United States (https://www.cdc.gov/drugresistance/biggest-threats.html) due to high rates of resistance to azithromycin and moxifloxacin. Additional treatment options are urgently needed. Nitroimidazoles, especially tinidazole, should be evaluated as alternative therapies to meet that need.

## MATERIALS AND METHODS

### Strains, media, and antibiotics.

M. genitalium strains used in this study included strains capable of axenic growth including the G37 type strain (35), M2282 and M2300 (36), and Sea-1 and Sea-2 (37). In addition, five recent Vero cell-dependent clinical strains cultured from men with urethritis were chosen as representatives of a variety of strain types with known resistance profiles for azithromycin, doxycycline, and moxifloxacin (9). Four of these strains (MEGA 601, MEGA 1166, MEGA 1256, and MEGA 1272) have been previously described (9); strain MEGA 1082 was cultured during the same study but has not been previously published. Axenic strains were grown in SP-4 (38), and Vero cell-dependent strains were grown in Vero cell cocultures in Eagle’s minimum essential medium (EMEM) supplemented with 10% fetal bovine serum, 6% yeast dialysate, and 25 mM HEPES, pH 7.2, as previously described (9). Antibiotics were purchased from Sigma and dissolved in water (moxifloxacin and doxycycline), dimethyl sulfoxide (DMSO) (nitroimidazoles), or 95% ethanol (azithromycin) and stored in aliquots at −20°C.

### Broth microdilution assays.

MIC values for M. genitalium strains capable of axenic growth were determined in broth microdilution assays (39). Briefly, M. genitalium frozen stocks were inoculated into SP-4 broth in 35-mm petri dishes and grown for 48 h at 37°C in 5% CO_2_ to obtain metabolically active log-phase bacteria. Adherent bacteria were scraped into the culture supernatant, passed through a 0.45-μm filter to remove aggregates, and then diluted in fresh SP-4 broth so that ~10^4^ CFU was contained in 0.1 mL. Twofold dilutions of antibiotics were prepared (in duplicate) in flat-bottom, 96-well plates, then the inoculum was added, and plates were incubated at 37°C/5% CO_2_ until control wells (no antibiotic) changed color from red to orange (due to fermentation of glucose, indicating growth), approximately 7 to 14 days. The outermost wells formed a moat consisting of M. genitalium inoculum at ~5 × 10^3^ CFU to minimize edge effects. The most dilute well with no color change was considered the MIC. DMSO alone, at a concentration corresponding to the highest concentration of drug, did not inhibit growth of M. genitalium in any experiments.

### Quantification of M. genitalium growth by qPCR.

To obtain precise measurements of inhibition, growth in broth microdilution assays was determined by qPCR. After assessing MIC endpoints by color change, 1/10 volume (20 μL) of Triton lysis solution (10% Triton X-100, 100 mM Tris HCl, pH 8, 10 mM EDTA) was added to each well and the plates were incubated at 95°C for 30 min. Lysates were diluted 1:10 in Tris-EDTA (TE), and then the number of M. genitalium genomes was quantified using a TaqMan qPCR method (40, 41). PCRs were performed in triplicate and compared to a quadruplicate standard curve of known genome concentrations. The drug concentration resulting in a 50% reduction (IC_50_) in M. genitalium genomes relative to untreated wells was calculated using a four-parameter logistic regression model.

### Vero coculture MICs.

MICs for Vero coculture-dependent clinical isolates were determined using a modification of previously published methods (9, 36). Briefly, dilutions of antimicrobials (in duplicate) were prepared in 96-well plates in 0.1 mL of EMEM containing 10% fetal bovine serum (FBS), 25 mM HEPES (pH 7.2), and 6% yeast dialysate and then inoculated with an 0.1-mL of suspension Vero cells (7 × 10^3^ cells/mL) and M. genitalium (~3 × 10^5^ genomes/mL). The M. genitalium inoculum for these clinical strains was diluted from frozen stocks of known concentration. Two identical plates were prepared for each strain and incubated for 21 to 28 days (strain G37 was incubated for 14 days), at which point the contents were lysed with Triton lysis solution and genomes were quantified by qPCR as described above.

### Testing synergism with doxycycline.

Synergism of metronidazole, secnidazole, or tinidazole with doxycycline was determined by the checkerboard broth microdilution method with nitroimidazole concentrations spanning 2-fold dilutions of 25 to 1.6 μg/mL and doxycycline spanning 2-fold dilutions of 4 to 0.016 μg/mL. FICI was calculated as previously described (10) with a FICI of ≤0.5 indicating synergism, a FICI of 0.5 to 4.0 indicating no interaction, and a FICI of >4.0 indicating antagonism.

### Time-kill experiments.

Time-kill experiments were performed similarly to those in the work of Waites et al. (42). M. genitalium G37 was grown to log phase, scraped, filtered, and diluted to ~1 × 10^5^ CFU per mL in l mL SP-4 broth containing 50, 25, 12.5, or 6.3 μg/mL of metronidazole, secnidazole, or tinidazole or 1, 0.5, 0.25, or 0.125 μg/mL of moxifloxacin. Controls included no antibiotic and DMSO solvent control at 0.5%, corresponding to the highest concentration of drug. At time zero and at daily intervals, the tubes were vortexed, and aliquots were diluted and spotted onto SP-4 agar plates in triplicate; colonies were counted after 2 to 3 weeks of incubation.

### Nitroimidazole-resistant mutants.

Nitroimidazole resistant mutants were isolated in two experiments. In the first method, M. genitalium strain G37C was serially passaged in SP-4 broth containing increasing concentrations of metronidazole and then single colony cloned on agar plates containing 50 μg/mL metronidazole. In the second method, >10^8^ cells of M. genitalium strain G37S (12) were plated directly onto SP-4 agar plates containing 25 to 50 μg/mL metronidazole, tinidazole, or secnidazole. Single colonies were cultured in SP-4 containing 50 μg/mL of the appropriate antibiotic. Resistant mutants were tested by broth microdilution for MICs to metronidazole, secnidazole, and tinidazole. Spontaneous resistance was measured by plating log-phase M. genitalium strain G37S on SP-4 agar containing 100 μg/mL of each antibiotic. Colonies arising after 3 weeks of incubation were counted and divided by total CFU on plain SP-4 agar.

### Whole-genome sequencing.

Total DNA from resistant mutants was isolated using the MasterPure complete DNA and RNA purification kit (Lucigen, Middleton, WI) according to the manufacturer’s instructions, except that tubes were mixed by inversion rather than vortexing to preserve high-molecular-weight DNA. Isolated DNA was suspended in nuclease-free water and sequenced using the Oxford Nanopore MinION sequencing device with a Rapid Barcoding kit on a 9.4.1 flow cell (Oxford Nanopore Technologies, Oxford Science Park, UK) according to the manufacturer’s instructions. Sequencing output was as follows: MtzR(A), 150 Mb of passed bases, 170× coverage, *N*_50_ of 12.0 kb; MtzR(B), 75 Mb of passed bases, 125× coverage, *N*_50_ of 16.1 kb; MtzR(E), 24 Mb of passed reads, 78× coverage, and *N*_50_ of 7.9 kb; and TdzR(A), 49 Mb of passed reads, 93× coverage, and *N*_50_ of 7.9 kb. Read files were processed using SAMtools (43), aligned to the G37 reference genome using GraphMap (44), and then manually examined for mutations using the Integrative Genomics Viewer (45).

### Growth in various oxygen environments.

Aerobic growth conditions consisted of growth in 5% CO_2_ and 95% oxygen (i.e., a tissue culture incubator). Microaerophilic conditions were achieved by incubating plates in a candle jar. Anaerobic conditions utilized an AnaeroGen sachet (ThermoFisher, Waltham, MA) in a sealed anaerobic jar with an anaerobic indicator. MICs for aerobic and microaerophilic conditions were read after 7 days of incubation; MICs for anaerobic conditions were read after 15 days due to slower growth.
